# Textile Antenna for Bio-Radar Embedded in a Car Seat

**DOI:** 10.3390/ma14010213

**Published:** 2021-01-04

**Authors:** Caroline Loss, Carolina Gouveia, Rita Salvado, Pedro Pinho, José Vieira

**Affiliations:** 1 FibEnTech Research Unit, Universidade da Beira Interior, 6201-001 Covilhã, Portugal; 2 Instituto de Telecomunicações, 3810-193 Aveiro, Portugal; carolina.gouveia@ua.pt (C.G.); ppinho@deetc.isel.pt (P.P.); jnvieira@ua.pt (J.V.); 3 Departamento de Eletrónica, Telecomunicações e Informática, Universidade de Aveiro, 3810-193 Aveiro, Portugal; 4 LabCom—Comunicação e Artes, Universidade da Beira Interior, 6201-001 Covilhã, Portugal; lrbss@ubi.pt; 5 Departamento de Engenharia Eletrónica, Telecomunicações e de Computadores, Instituto Superior de Engenharia de Lisboa, 1959-007 Lisboa, Portugal

**Keywords:** textile antenna, dielectric substrate, bio-radar, vital signs, non-contact measurements

## Abstract

A bio-radar system is presented for vital signs acquisition, using textile antennas manufactured with a continuous substrate that integrates the ground plane. Textile antennas were selected to be used in the RF (Radio Frequency) front-end, rather than those made of conventional materials, to further integrate the system in a car seat cover and thus streamline the industrial manufacturing process. The development of the novel substrate material is described in detail, as well as its characterization process. Then, the antenna design considerations are presented. The experiments to validate the textile antennas operation by acquiring the respiratory signal of six subjects with different body structures while seated in a car seat are presented. In conclusion, it was possible to prove that bio-radar systems can operate with textile-based antennas, providing accurate results of the extraction of vital signs.

## 1. Introduction

The vital signs acquisition through contactless means is a hot topic in the scientific community because it can be a promising tool to support healthcare and enhance smart systems. The so-called Bio-Radar system is a radar-based technology capable of acquiring the respiratory and cardiac signals without contact sensors.

**Figure 1:**
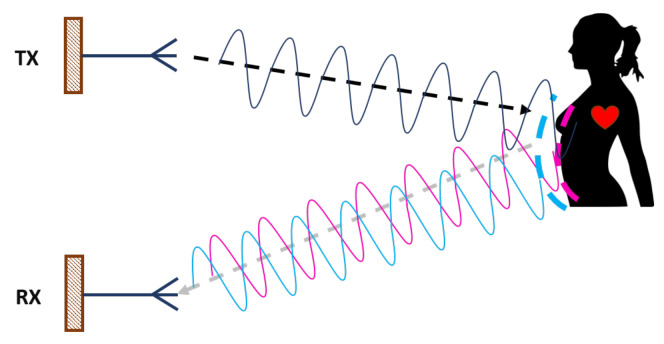
Bio-radar working principle.

Generally, the bio-radar systems are composed of an antenna for transmission (TX), which focuses the energy towards the subject chest-wall, and another antenna for the reception (RX) to acquire its reflection. The received signal is a phase modulated version of the transmitted one. This modulation is triggered by the chest-wall motion that changes the travelled distance of the electromagnetic waves [1]. The working principle is depicted in Figure 1.

The antenna design plays a crucial role in the performance of the bio-radar system, and it should be optimized to maintain a signal-to-noise ratio (SNR) at a superior level [2]. Furthermore, the antenna is also the most flexible component of the system since it can be designed according to the specific requirements of the targeted application. Besides, it can be manufactured using different materials, and thus, it enables the integration of the full system in different monitoring environments. Considering the vehicular applications, the bio-radar can be used to monitor the vital signs of the driver, reducing the probability of accidents due to sudden disease or to the fatigue state.

Several works have been reported, where different radar front-ends were integrated inside the car cabin for monitoring purposes. For example, in [3], the authors presented a Continuous Wave (CW) radar, operating at 24 GHz to detect the subject presence, namely babies. This prototype was located on the car roof, above the baby seat. In this work, antennas were designed to enhance the detection range, and algorithms based on vital signs extraction were developed to detect the presence of the subject, even if they are sleeping. Systems to monitor the driver’s vital signs were also presented, as in [4], where a system based in a Software Defined Radio (SDR) front-end is integrated into a car seat, inside its structure to be more specific. Two pairs of antennas are disposed in the back and inferior seat part of acquiring vital signs from the back and the subject’s legs, respectively. This system operates using a dual harmonic approach at low frequencies, namely 475 MHz and 950 MHz. Thus, the antennas should be in contact with the human body in order to acquire vital signs through body coupling means. In [5], other work using coupling antennas to acquire the driver’s vital signs was presented. An Ultra-Wide Band (UWB) radar was implemented inside the car seat structure, using commercial radar front-ends with coupled antennas also located in the back area.

All the works herein mentioned used commercial front-ends with antennas made using conventional substrates, which were mostly in direct contact with the subject. The direct contact can affect the results due to the subject’s motion, and the antennas could get damaged considering a long-term perspective. Thus, the exact location of the antennas should be selected with care to avoid their abrasion. Moreover, the use of antennas made with conventional substrates near to the body can be uncomfortable due to the hardness of the material.

The usage of textile antennas can be an excellent solution to integrate the bio-radar in near body applications, such as in the vehicular scenario. Indeed, textile antennas have already been proposed for wearable radar applications, more specifically for motion sense and target detection [6,7], by integrating antennas into the subject’s clothes.

To ensure a low profile and the unobtrusive integration of the bio-radar into the car seat, the antennas must be thin, lightweight, robust, and easy to maintain. In this sense, planar textile antennas are a promising solution because this type of antenna combines low profile characteristics and can also be adaptable to any surface. Such antennas are usually formed by assembling conductive (patch and ground plane) and dielectric (substrate) layers. Thus, to develop planar textile antennas, the knowledge of the electromagnetic properties of textile materials is crucial [8].

Beyond the selection of textile materials to develop antennas, the manufacturing technique must also be chosen carefully to ensure the mechanical stabilization of the antenna, since the textile materials are highly deformable. Although the patch of the textile antenna can be easily cut, laminated [9,10], screen-printed [11,12] or embroidered [13,14] by industrial machines, the conception of a good industrial substrate, that meets all the mechanical and electromagnetic requirements for textile antennas, remains a challenging task. Even though, the integration of the antenna into a single textile structure can contribute to an easier and faster industrial manufacturing process, and can also reduce the incurrences due to the hand-made manufacturing process. This concept was already presented in [15,16,17].

In [17,18], the authors have presented a 3D Integrated Microstrip Antenna, which is woven into a 3D orthogonal fabric. This microstrip antenna was developed to work as a radar L-band (1.5 GHz) for aerospace applications. It was made using copper yarns for the conductive parts and aramid (Kevlar 129) yarns for the dielectric layer. Despite the measured resonance frequency was shifted to a frequency higher than planned (1.8 GHz), this has proved that the antenna design can be integrated into unique material composed of multiple layers.

Later, in [19], the authors presented a Substrate Integrated Waveguide (SIW) textile antenna, also for radar applications, operating at 77 GHz. In this work, the antenna was designed taking into account the characteristics of the conductive and dielectric yarns, to be manufactured using industrial weaving textile machinery, avoiding hand-made procedures. For the dielectric substrate, a polyester yarn was considered, with a $\varepsilon_{r}=3.2$. For the conductive parts, copper filament yarns with a conductivity of 58 MS/m were used. This novel antenna has worked at the proposed frequency, showing good agreement between the simulated and the measured results.

Combining all the mentioned concepts, this work presents the development of antennas for bio-radar, using planar printed textile antennas manufactured with a novel textile material that integrates the dielectric substrate and the conductive ground plane in a single sheet. This manuscript is divided as the following: Section 2 presents the materials and methods used to manufacture and characterize the novel textile substrate. Further, Section 3 describes the design process of textile antennas using the novel substrate, considering the bio-radar system operation mode. Section 4 presents some experiments that have been conducted to validate the usage of textile antennas in the bio-radar system. This section is divided in two parts: first, a comparison test was performed, where the respiratory signal was acquired using a textile and conventional substrate antennas. The same test was then repeated using only textile antennas, where six subjects with different physiognomies were monitored. Later, in Section 5, a brief discussion of the results is presented. The main conclusions are reported in Section 6.

## 2. Material and Methods

**Figure 2:**
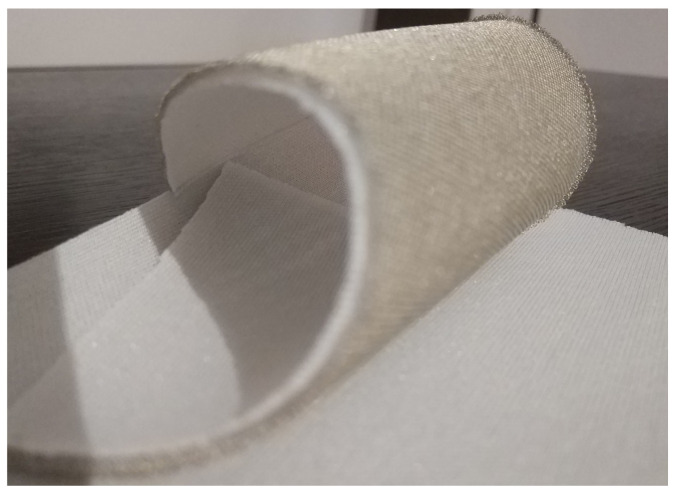
Continuous Substrate Integrating the Ground Plane-3D Weft knitted spacer fabric (white) with an integrated conductive layer (gold).

**Figure 3:**
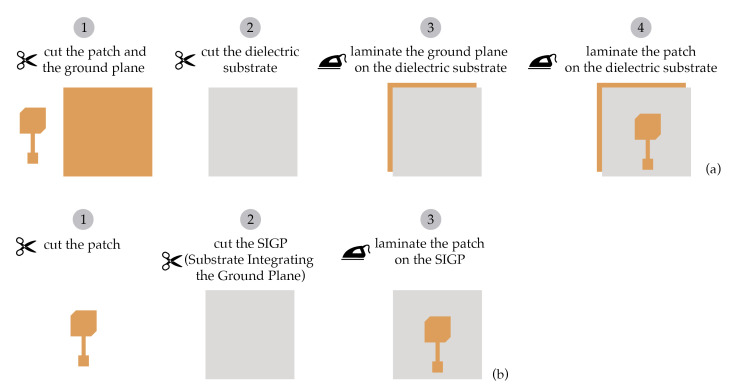
Comparison between the step-by-step of the laminating manufacturing technique, where (**a**) is the typical process used to assembly textile antennas, and (**b**) is the process to assemble textile antennas that uses Substrate Integrating the Ground Plane (SIGP) as dielectric substrate.

In this paper, a novel substrate is proposed, namely the Substrate Integrating the Ground Plane (SIGP), based on the weft-knitted spacer fabric. The SIGP thus is a novel textile material that integrates the dielectric substrate and the conductive ground plane in a single sheet, as presented in Figure 2. The integration of the ground plane into the substrate can decrease the losses and the mismatches caused by the hand-made manufacturing process [10], since it reduces one step of the laminating procedures, as one can see in Figure 3. The continuous ground plane shields the antenna radiation, ensuring that the human body is exposed only to a very small fraction of the radiation.

### 2.1. Development of a Continuous Substrate Integrating the Ground Plane

**Figure 4:**
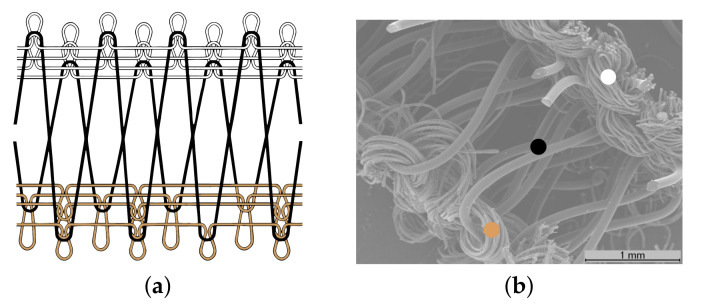
Scheme of the SIGP: (**a**) Weft-knitting structure diagram based on [18], where the white, black and brown yarns correspond to the dielectric, spacer and conductive yarns, respectively; (**b**) SEM image of SIGP, with 35X amplification.

The SIGP is a weft-knitted spacer fabric, produced in a double circular machine, the V-LEC6BS Knitting Machine (Monarch Knitting Machinery Ltd., Leicester, UK), which has a routable needle cylinder and a needle dial, capable to produce a 3D weft-knit with differentiated faces. The SIGP was manufactured at Borgstena Textile Portugal Lda., following the manufacturing process of the Patent US 6779369 B2 [18]. In this work, the patented method was used in an innovative approach to manufacturing a weft-knitted fabric whose material faces have different physical properties, namely one conductive and one dielectric layer. Figure 4 presents the knitting structure and the Scanning Electronic Microscope (SEM) image of the transverse cross-section of the SIGP.

Based on the weft-knitting structure presented in Figure 4, the SIGP was developed using the following yarns:

- Dielectric substrate (front side layer-white): a 100% Polyester yarn;
- Spacer yarn (interior layer-black): a Monofilament FH yarn, 100% Polyester (PolyEthylene Terephthalate, PET), 225 dtex, produced by Monosuisse AG (Emmenbrücke, Switzerland);
- Conductive layer (back side layer-brown): a Shieldex^®^ 117/17 dtex Z-turns HC+B yarn, produced by Shieldex Trading (Palmyra, PA, USA).

The Shieldex^®^ 117/17, is a filament yarn composed by 117 high tenacity Polyamide 6.6 filaments and 17 twisted filaments coated with 99% of pure silver. The linear mass of this yarn is 141 dtex, and it presents a resistivity <500Ω/m (these values were given by the manufacturer). Besides the electrical conductivity, the choice of the yarn was based on their linear mass (dtex), which must be suitable to work with the V-LEC6BS Knitting Machine. All the yarns are deliberately composed of synthetic fibres, aiming at a low interaction with moisture and thus minimizing its effect on the electromagnetic performance of the materials [19,20].

Furthermore, another spacer knit sample using the polyester yarn on both sides was produced to serve as a control material. This sample aims to serve as a reference to analyse the influence of the integrated ground plane in the dielectric behaviour of the material. The structural parameters and the electromagnetic properties of both samples will be described in the following subsections.

#### 2.1.1. Characterization of the Structural Parameters

**Figure 5:**
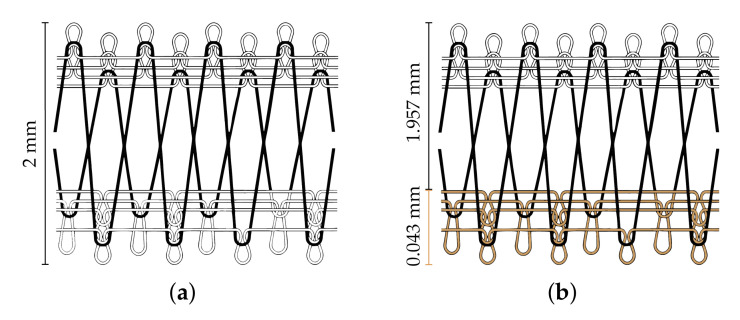
Difference of thickness between samples: (**a**) reference spacer knit and (**b**) SIGP. Where the white, black and brown yarns correspond to the dielectric, spacer and conductive yarns, respectively.

The thickness of the samples was measured using the KES-F—3 Compressional Tester of Kawabata’s Evaluation System for Fabrics [21]. The tests were performed under controlled environmental conditions, namely $20{\;}^{\circ }$C$\;\pm \;2^{\circ }$ and 65%±2% of Relative Humidity (RH), and five samples of each spacer knit were tested, to determine the total thickness (see Figure 5a). The average and standard deviation values are presented in Table 1. The thickness of the conductive layer of SIGP (see Figure 5b) was measured through SEM image analysis. The superficial porosity was also calculated through the image analysis method, using the DiameterJ tool [22], of the ImageJ software image analyser [23]. To calculate the superficial porosity, SEM images were used, with 35× amplification. Figure 5 illustrates the difference of thickness of the knit structure before (reference spacer knit) and after (SIGP) the integration of the conductive layer. Table 1 summarises the structural parameters of the SIGP and the reference spacer knit.

#### 2.1.2. Characterization of the Electromagnetic Properties

##### Conductivity

Fabrics are planar materials and, therefore, their electrical behaviour may be quantified by the surface (or sheet) resistance ($R_{s}$) and characterized by the surface (or sheet) resistivity ($\rho_{s}$). The conductivity of the integrated ground plane was characterized following the ASTM Standard F 1896—Test Method to Determine the Electrical Resistivity of a Printed Conductive Material [24]. In this standard process, first, the sheet resistance ($R_{s}$) is measured using a resistance measuring electronic device. In this case, an Agilent HP 34,401A Multimeter was used, and the results are given in Ω. Then, considering the sample dimensions, the sheet resistivity was calculated. Since the $\rho_{s}$ results are given in Ω/square, the conductivity was further calculated by Equation (1),

(1) $$\sigma =\frac{1}{\left(\rho_{s}\cdot h\right)}$$

where σ is the conductivity, $\rho_{s}$ is the measured sheet resistivity and *h* is the thickness of the material. In this case, only the thickness of the conductive layer (0.043 mm, see Figure 5b) was considered.

Five samples of SIGP were measured under the environmental conditions of $20{\;}^{\circ }$C$\;\pm \;2^{\circ }$ and 65%±2% of RH. Table 2 presents the test parameters and results.

##### Permittivity and Loss Tangent

In order to characterize the dielectric properties of the SIGP, the Microstrip Resonator Patch Method was used [25]. This method consists on designing a microstrip patch antenna using an estimated $\varepsilon_{r}$ value and calculating the real $\varepsilon_{r}$ based on the shift of the measured resonant frequency of the antenna under test.

**Figure 6:**
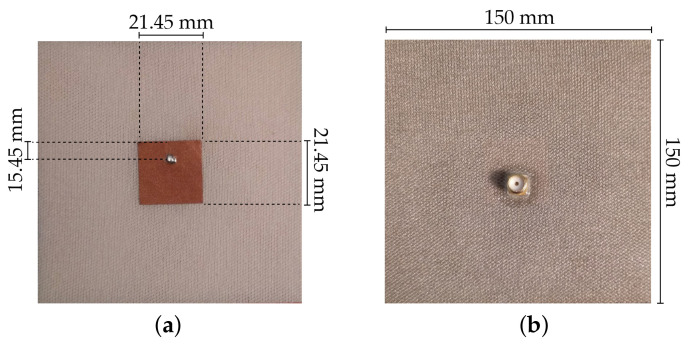
Textile microstrip patch antenna used to characterize the dielectric properties of SIGP: (**a**) front and (**b**) back.

Thus, one microstrip patch antenna was designed to resonate at 5.8 GHz, using the SIGP substrate. The antenna was simulated in the CST Microwave Studio 2017 full-wave simulator, using the estimated values of $\varepsilon_{r}=1.10$ and tanδ=0.006. These estimated values were based on a previous dielectric characterization of different types of 3D fabrics [26]. For manufacturing the patch, a Pure Copper Polyester Taffeta Fabric (PCPTF) (Less EMF Inc., Latham, NY, USA) was used, with 0.08 mm of thickness and conductivity equal to 62.5 kS/m. Figure 6 presents the design and the dimensions of the textile microstrip patch antenna.

**Figure 7:**
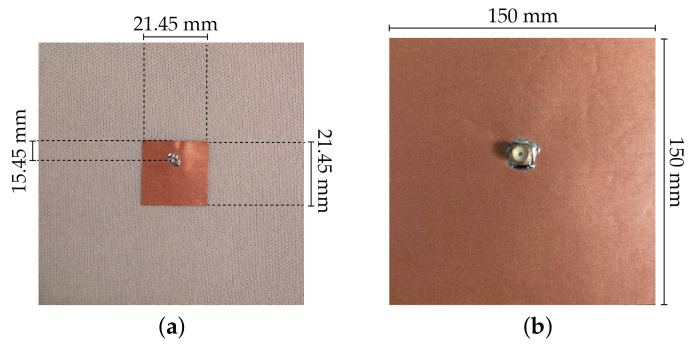
Textile microstrip patch antenna used as control sample: (**a**) front and (**b**) back.

For control purposes, the same designed antenna for 5.8 GHz was also manufactured using the reference spacer knit as dielectric substrate and the PCPTF for both patch and ground plane. The manufactured control antenna is depicted in Figure 7.

The textile microstrip patch antennas were manufactured with a laminating technique. It consists of assembling the components with the thermal adhesive sheet through an ironing operation. A 100% polyamide thermal adhesive sheet (JAU Têxteis, Serzedo, Portugal), with 0.01 mm of thickness, was used. The laminating process was made using an industrial ironing press, under 10 bar, at 200 ${}^{\circ }$C, during 6 s, without steam. All parts of the antenna were cut by a laser cutting machine (Jinan G. Weike Science & Technology Co. Ltd., Shandong, China) to ensure geometrical accuracy.

To feed the antennas, a SubMiniature version A (SMA) connector was used. The knitted structure of the SIGP, presenting large pores, does not allow the full welding of the SMA connector, as it has been previously performed in making textile antennas. In this case, the SMA connector was glued to the integrated ground plane using a conductive glue Elecolit^®^ (Panacol-Elosol GmbH, Steinbach, Germany) and an extra coating of Slow-Cure^™^ Epoxy (Bob Smith Industries Inc., Atascadero, CA, USA) was applied in order to ensure the mechanical stability. In the patch side, the SMA connector was welded on the conductive fabric.

**Figure 8:**
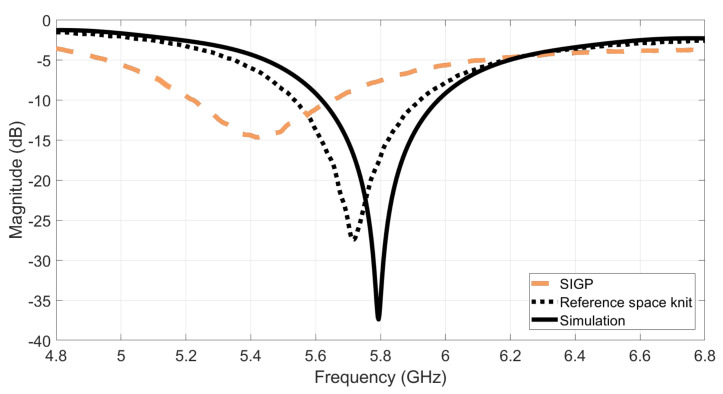
Simulated and measured $S_{11}$ of the textile microstrip patch antennas designed for the dielectric characterization.

After manufacturing both antennas, their performance was evaluated by measuring the $S_{11}$ parameter, using a Vector Analyzer Network (VNA). The $S_{11}$ parameter is one of the scattering parameters that describes the input–output relationship at each port, or terminal, in an electromagnetic system. The $S_{11}$ defines the relationship between the injected signal into the antenna and the reflected signal at that specific port, with all the others matched [27]. The reflection of the injected signal occurs due to impedance mismatch. A minimum $S_{11}$ parameter is reached when there is a perfect match between the antenna impedance and the feed line. To determine the bandwidth of an antenna, the −10 dB return loss value is used. Figure 8 presents the obtained results.

As one can see on Figure 8, the control antenna which was made with the reference spacer knit and the PCPTF, presents a closely agreement with the simulated $S_{11}$. This result was expected, as the simulation was made using the conductivity value of the PCPTF for both of the conductive parts (patch and ground plane). It is also important to note that during the simulation process an estimated values of permittivity and loss tangent was used, which can lead to small frequency deviations. Besides, the manual process of laminating the patch also can introduce some inaccuracies. Despite the small frequency deviation, the estimated values of permittivity and loss tangent characterizes well the dielectric behaviour of the reference spacer knit.

The SIGP antenna presents an acceptable performance within the evaluated bandwidth. However, a frequency deviation (5.42 GHz) can be observed when comparing its $S_{11}$ to the measured result of the antenna manufactured using the reference space knit (5.72 GHz), and to the simulated one (5.8 GHz). As textile materials present a quite narrow range of relative permittivity values, it is therefore their thickness, which values may present much larger variations, that will mainly determine the bandwidth as well the input impedance of the antenna and so its resonance frequency [27,28]. As one can see in Figure 5, the integration of the ground plane on the same sheet of the substrate, reduces the thickness of the dielectric substrate, thus changing the Q-factor of the antenna as described in [27,28]. Indeed, during the simulation process, a total thickness of the substrate equal to 2 mm was considered for both materials. However, the SIGP fabric have already included the ground plane, whose thickness is 0.043 mm, and thus its dielectric substrate is thinner than 2 mm (see Figure 5b). On the other hand, the control antenna was made using the reference spacer knit as dielectric substrate, whose thickness is 2 mm, and the ground plane was a separated layer, made using PCPTF, with thickness is equal to 0.08 mm.

Based on these results, first, a new simulation of the SIGP antenna was made using the corrected thickness value (being *h* = 1.957 mm for the dielectric substrate and *h* = 0.043 mm for the ground plane). Then, as the integration of the conductive layer on the same sheet of the dielectric substrate also change the electromagnetic characteristics of the material, a new permitivitty characterization was performed. In this way, as the resonance frequency of the microstrip patch antenna is inversely proportional to the square root of the permittivity of the substrate [27], the permittivity value was increased in the simulation environment until the new simulated $S_{11}$ be in agreement with the measured one. In this way, the new value of $\varepsilon_{r}=1.3$ was obtained.

**Figure 9:**
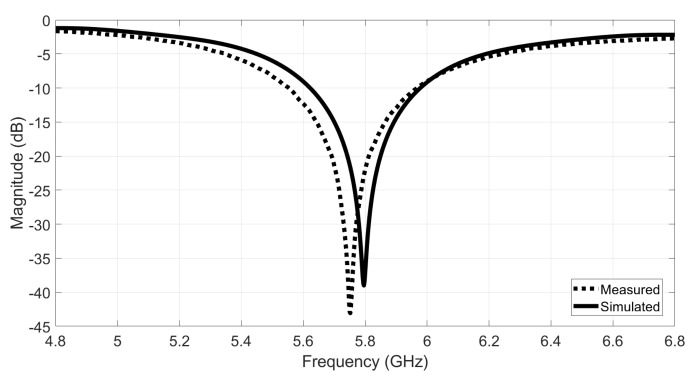
Simulated and measured $S_{11}$ control antenna design for the new $\varepsilon_{r}$.

In order to guarantee the antenna’s parameters accuracy, a new SIGP antenna was simulated considering the new $\varepsilon_{r}=1.3$ and built. Figure 9 shows the measured $S_{11}$ of the new manufactured SIGP antenna, where it is possible to verify that despite a small frequency deviation (5.75 GHz) the measured results are closed to the simulated ones. Assuming the $\varepsilon_{r}=1.3$ as the real value of the substrate, one may estimate the conductivity of the ground plane of SIGP equals to 52 kS/m, which validates the results measured in the previous subsection.

## 3. Development of Textile Antenna for Bio-Radar

**Figure 10:**
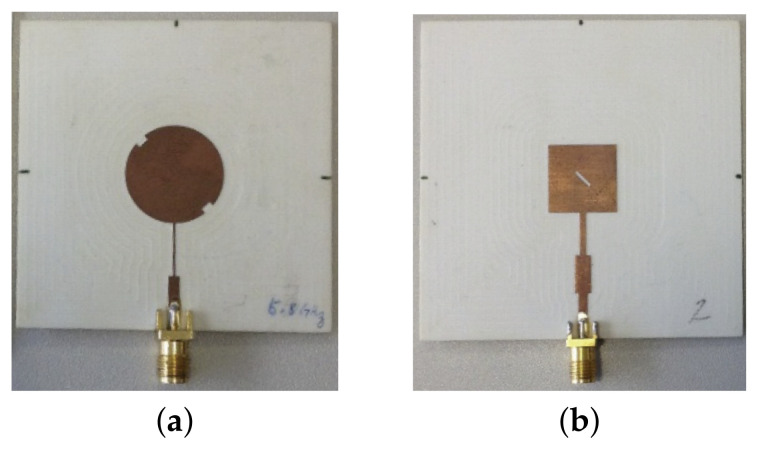
Left-hand circularly polarized (LHCP) antennas made using conventional substrate for 5.8 GHz: (**a**) circular patch antenna [29] and (**b**) squared patch antenna.

After the structural and electromagnetic characterization, the SIGP was used to design and build two textile antennas for bio-radar, operating at 5.8 GHz. To evaluate the performance of the textile antennas, a comparison is going to be made using two antennas with a conventional rigid substrate. These antennas are left-hand circularly polarized (LHCP) microstrip patch antenna, being a circular [29] and a square patch, respectively. These antennas operate at 5.8 GHz, and they were made using a well-know substrate called Rogers RO4360G2^™^ (Rogers Corporation, Chandler, AZ, USA). This high-frequency laminate has the dielectric part composed of glass-reinforced and hydrocarbon ceramic-filled thermoset materials, with $\varepsilon_{r}=2.55$, tanδ=0.0038 and 0.78 mm of thickness. The conductive layers are made of copper, with 17.5 μm of thickness and conductivity equals 58,000,000 S/m. Figure 10 presents the antennas used for comparison purposes, with Rogers RO4360G2^™^ substrate.

These served as a basis for the textile antennas design. Therefore, their design was replicated for the new textile material. It is important to note that a simpler design was selected for the squared patch, to streamline the model and simulation process. The usage of microstrip feeding lines was selected in order to obtain a proper comparison.

### 3.1. Design of Textile Antennas

**Figure 11:**
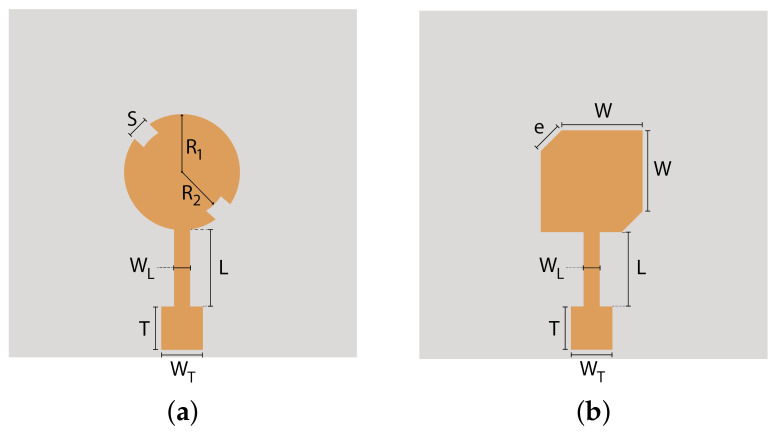
LHCP antennas made using SIGP for 5.8 GHz: (**a**) circular patch antenna and (**b**) squared patch antenna.

The textile antennas were simulated on CST Microwave Studio software, using the SIGP as dielectric substrate and the PCPTF for the patch. Since it is intended to replicate the antennas presented in Figure 10 with a conventional substrate, single patches were designed equally with circular polarization, where one is a circularly-shaped patch with slots (Figure 11a), and the other is a square patch with chamfered corners, (Figure 11b).

Similarly to the conventional substrate antennas presented in Figure 10, these antennas were fed using a feeding line, which is composed by a quarter wavelength transformer identified by line *L* and a 50 Ω line identified by *T*. Both textile antennas are 70×70 mm and their final dimensions are presented in Table 3.

### 3.2. Measured Parameters of Textile Antennas

**Figure 12:**
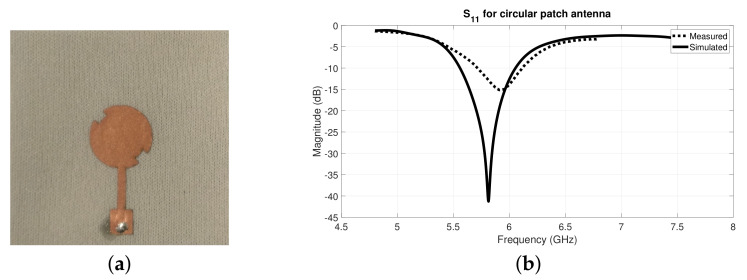
Circular patch textile antenna for 5.8 GHz: (**a**) manufactured antenna and (**b**) $S_{11}$ results.

**Figure 13:**
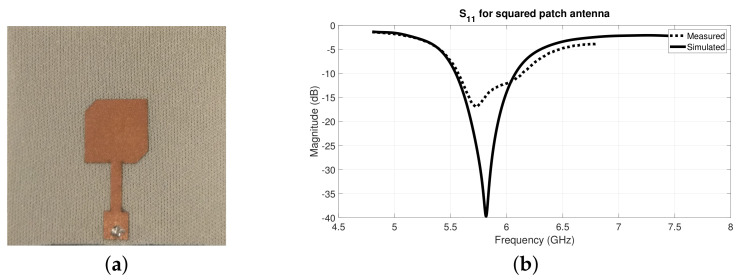
Squared patch textile antenna for 5.8 GHz: (**a**) manufactured antenna and (**b**) $S_{11}$ results.

After optimizing the textile antennas in the simulation environment, they were also manufactured using the laminating technique, as described in Section 2.1.2. Further, the $S_{11}$ parameter of both textile antennas was measured using a VNA. Figure 12 and Figure 13 presents the manufactured antennas and their results.

**Figure 14:**
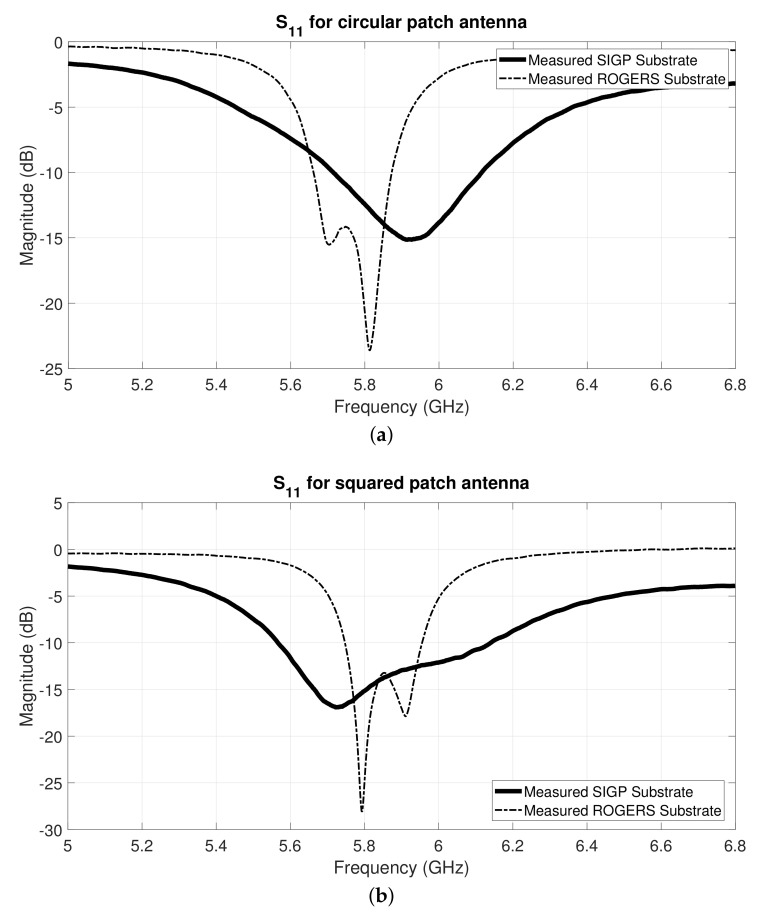
Comparison of the measured $S_{11}$ of textile and rigid antennas. (**a**) Circular patch antennas and (**b**) squared patch antennas.

Through the analysis of the $S_{11}$ graphs from Figure 14b and Figure 12b, for the squared and circular patch respectively, it is possible to observe that the measured results are slightly shifted in frequency, in relation to the simulated ones. Nonetheless, acceptable $S_{11}$ values were obtained for 5.8 GHz, namely −15.15 dB for the squared patch and −12.43 dB for the circular patch. These antennas have several losses on their efficiency since textile substrate material is heterogeneous and highly porous, hence small deviation in the measured results were expected.

For performance comparison purposes, Figure 14 presents the measured $S_{11}$ results of the textile antennas and the antennas manufactured with conventional (rigid) substrate, as the ones presented in Figure 10. However, it is important to note that for the squared patch case, this comparison might not be direct as for the circular patch ones, since the patch design is not exactly the same.

The obtained results for the textile antennas are similar to the obtained ones for the conventional substrate antennas. Both textile and conventional substrate antennas present a higher $S_{11}$ magnitude. This might be explained by the selected feeding method, where both antennas use quarter wavelength transformers followed by 50Ω lines. This feeding method is more susceptible to losses and bandwidth limit [27].

Beside the $S_{11}$, the radiation pattern was also measured and it is presented in

**Figure 15:**
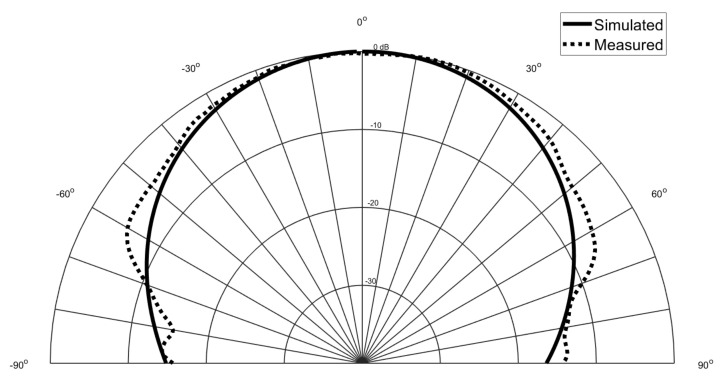
Simulated and measured radiation pattern of the circular patch antenna.

**Figure 16:**
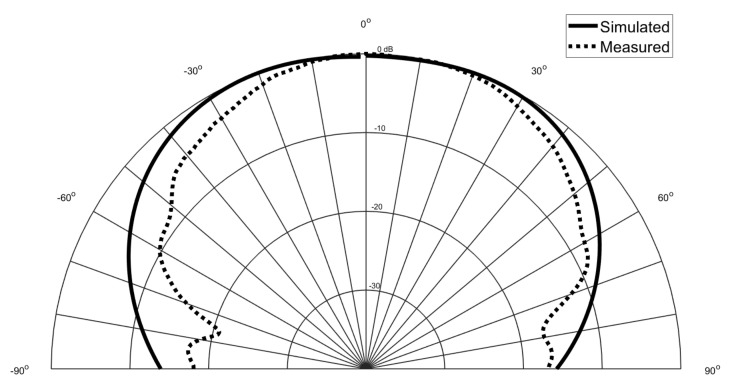
Simulated and measured radiation pattern of the squared patch antenna.

Figure 15 and Figure 16. The radiation pattern was measured inside an anechoic chamber and the Phi = $0^{\circ }$ was the selected plane. It is possible to observe that once again the achieved results are in agreement with the simulated ones.

## 4. Practical Implementation

To evaluate the capability of the textile antennas to acquire the respiratory signal accurately, several experiments were conducted using the bio-radar system. The bio-radar consists of a real-time measuring system implemented with the LabVIEW software [30]. The respiratory signals are acquired using the two manufactured textile antennas and an RF front-end based on an SDR system. The radar front-end used was the USRP B210 board from Ettus Research™, using the CW operation mode, with the carrier frequency equal to 5.8 GHz and a transmitting power equal to −9 dBm.

First, the exact location of the antennas had to be chosen considering the target population and the chest-wall expansion. Thus, the side lumbar support was the selected location since it can measure the side expansion of the chest-wall. In addition, this location was selected considering the integration of the bio-radar into the seat car. Thus, the respiratory signal was acquired using the developed textile antennas, previously described in this work and the conventional substrate antennas presented in Figure 10. Finally, considering a fixed position of the antennas in the selected car seat spot, the respiratory signals of six subjects were acquired, testing the acquisition limits for different body physiognomies. All the experiments were performed in a laboratory environment, and they are explained in detail in the following subsections.

### 4.1. Textile Antenna Validation Test

**Figure 17:**
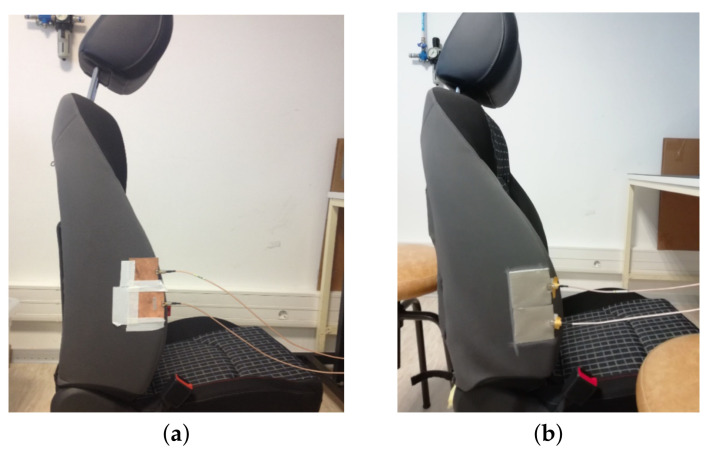
Position of the transmission (TX) and reception (RX) antennas, in the car seat, to acquire the respiratory signs: (**a**) conventional substrate antennas and (**b**) textile antennas.

A preliminary test was carried out to verify if it was possible to acquire respiratory signals using textile antennas located in the side lumbar support. For this purpose, the respiratory signal of one subject was captured using both textile antennas and conventional substrate antennas, and their performances were compared. Figure 17 illustrates the position of the antennas in the car seat to acquire the respiratory signs.

**Figure 18:**
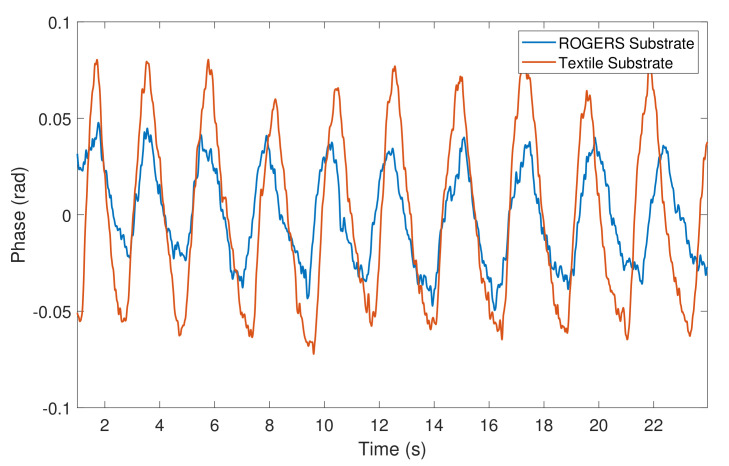
Comparison of the respiratory signals acquired using conventional and textile substrate antennas.

Before starting the measurements, the subject was asked to breathe normally during the full test. The subject’s arms were placed on a table located in front of the car seat to simulate the driver’s posture. Figure 18 shows the respiratory signals acquired using both antennas.

Observing Figure 18, it is possible to see that the textile antennas have the same capability to detect the respiratory signals when compared with a conventional substrate antenna. However, it is important to note that even preserving the same monitoring conditions, those respiratory signals were not acquired at the same time, therefore the differences in the SNR are not comparable.

### 4.2. Bio-Radar Validation

**Figure 19:**
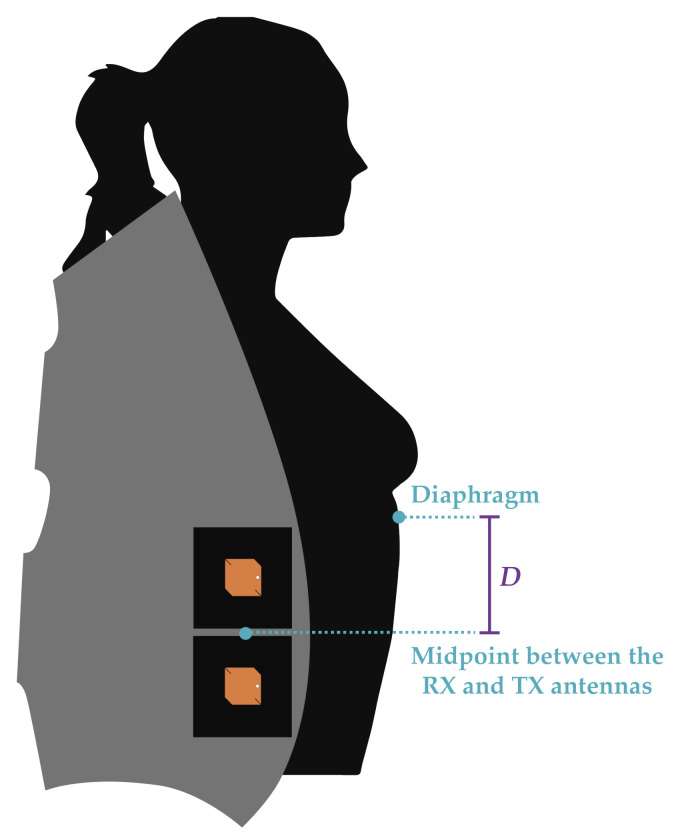
Set-up for the six subject experiment, side view.

Being the textile antennas suitable to work on the bio-radar, an experimental test with six different subjects was performed. This test aimed to verify if it is possible to acquire the respiratory signals of subjects with different body structures, while the position of the antennas was the same for every subject. Table 4 summarises the physical description of the subjects under test, according to their position in the car seat depicted in Figure 19.

As physical parameters, the height and chest-wall width of each subject were considered, as well as the distance *D*. As one can see on Figure 19, the *D* is the distance between the diaphragm and the midpoint between the RX and TX antennas. The distance *D* is the most critical parameter since it can vary largely regardless of the subject’s height. Furthermore, this measure shows which expansion is the radar evaluating, which can vary between the belly motion or the chest-wall motion. For people with larger chest cavities, the radar could also measure the back motion, rather than the chest-wall.

**Figure 20:**
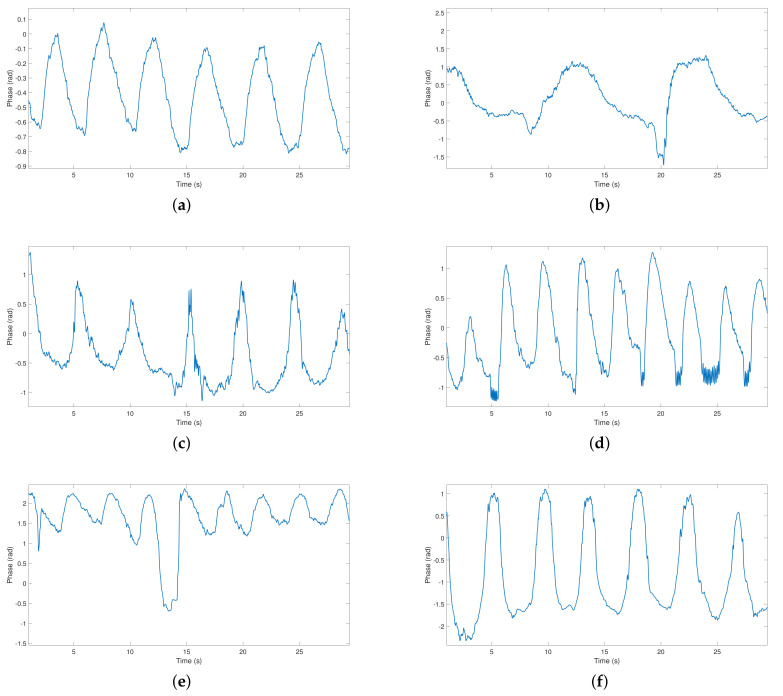
Respiratory signals of six subjects detected using textile antennas: (**a**) subject 1, (**b**) subject 2, (**c**) subject 3, (**d**) subject 4, (**e**) subject 5, (**f**) subject 6.

The acquired signal was captured during 30 s in a laboratory environment. Figure 20 presents the acquired respiratory signals of each subject under test.

As one can see in Figure 20, it is possible to detect the respiratory signals of all subjects regardless of their physical structure or *D* value, which means that the signal can be acquired even if the detection points are not optimal. Since the signals were captured at the same time interval (30 s), it is possible to distinguish diverse characteristics among the detected signals, such as the respiratory rate. For example, the signal from subject 2 has a low respiratory rate, and subject 4 breathes at a higher rate. Other breathing patterns can also be identified, for example, subject 5 had laughed after 15 s of the experiment, which is represented by a sudden slope on the waveform.

**Figure 21:**
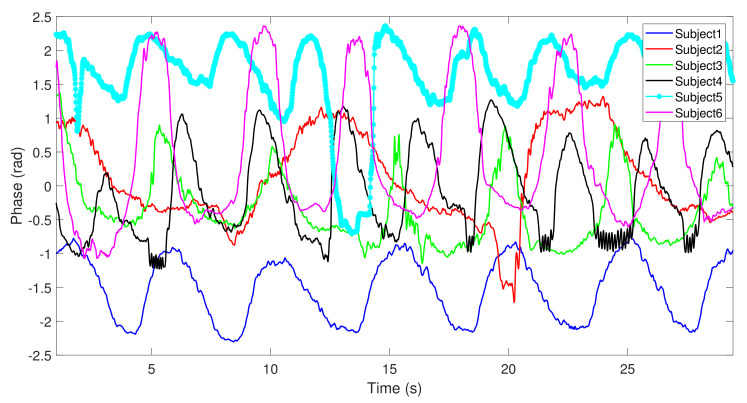
Respiratory signals of six subjects with a fixed axis.

As the positioning of the antennas was adjusted to the optimal detection point for the first subject, but was not changed between subjects, signals present different peak-to-peak amplitudes, different mean values, and different noise levels, when compared with the first acquired signal. Figure 21 presents the same six signals, but keeping the same axis scale. Herein, it is possible to see large differences in the SNR between these signals, as well as visualize other waveform disparities relatively to the first acquired signal.

## 5. Discussion

The integration of the textile ground plane into the surface of the textile substrate, all in one fabric that has multiple layers, is the first step to the industrialization of the textile microstrip patch antennas. The knitting spacer technologies are particularly suited for this purpose, allowing the production of thick fabrics with different surfaces. The weft-knitted spacer fabrics with an integrated conductive layer, herein developed and presented, have shown being a promising approach.

The integration of the ground plane on the surface of the substrate changes the dielectric properties of the substrate material, increasing its dielectric constant value. This is mainly due to the decrease in the thickness of the substrate. Besides that, the SIGP presented good electromagnetic properties, thus it is suitable for the development of textile microstrip patch antennas for bio-radar.

Through the results obtained in the proof of concept tests, it is possible to conclude that the textile substrate antennas can detect the respiratory signals similarly to the conventional substrate antennas, which are commonly used. The last test also proved the efficiency of the bio-radar system, for different subjects independently of their physiognomies, if the antennas location remains unchanged. However, the location of the antennas must always be selected with care since the detection of vital signs can be hampered for subjects with body structures overly larger or smaller, due to the loss of chest-wall illumination.

Besides, for a real scenario application, an appropriate feeding method should be selected, taking into consideration the connector weight and the force applied by the feeding cables. Since the textile substrate of antennas is flexible and highly deformable, their operation mode can become affected if the connector used is too heavy to guarantee the textile stability and hence cause its deformation. In these cases, lighter connectors should be selected.

## 6. Conclusions

In this manuscript, the manufacturing and characterization process of a novel textile substrate integrating the ground plane was presented. This material was used for the development of a bio-radar system to acquire the respiratory signal remotely. The manufactured textile antennas were measured and have presented acceptable $S_{11}$ values, below −12 dB. Their usage was validated by comparing the acquired respiratory signals to the ones acquired with conventional substrate antennas.

Furthermore, a performance evaluation was done by acquiring the respiratory signal of six subjects with different body structures, without changing the location of the antennas between subjects. It was possible to acquire the vital signs for every case and additionally to identify different breathing patterns.

Nonetheless, the development of textile antennas for this scope should be done with care. The characterization of the dielectric properties should be done considering the frequency of operation at hand, and thus avoiding frequency shifts in the practical $S_{11}$ measures. Additionally, the antenna’s bandwidth should also be increased, encompassing errors resulting from the manufacturing process.

In general, the validation described herein leads one to conclude that the textile substrate integrating the ground plane is very promising for boosting the industrial fabrication of microstrip patch textile antennas and their mass production. Thus, the textile antennas can enhance bio-radar integration into any application.

## Figures and Tables

**Table 1:** Structural Parameters of the SIGP and of the reference spacer knit.

	Total Thickness (mm)	Thickness of Conductive Layer (mm)	Density (Number of Loops/cm)				Superficial Porosity (%)	
			Front Side		Back Side			
			Wales	Courses	Wales	Courses	Front Side	Back Side
SIGP	2 ±0.01	0.043	18	13	18	13	45.04	45.71
Reference	2 ±0.01	-	18	13	18	13	45.02	45.01

**Table 2:** Electrical properties of SIGP.

Dimensions of the Samples (mm)		Sheet Resistance (Ω/sq.)	Conductivity (kS/m)
Length	Width		
30	60	0.428 ±0.03	54

**Table 3:** Final dimensions of both optimized textile antennas [mm].

Circular Patch Antenna							Squared Patch Antenna					
**R1**	**R2**	**s**	**L**	**T**	$w_{L}$	$w_{T}$	**e**	**W**	**L**	**T**	$w_{L}$	$w_{T}$
11.60	9.35	4.10	14.00	8.85	3.00	8.30	5.50	20.40	14.50	8.50	3.65	8.00

**Table 4:** Physical description of the subjects under test.

Subject N${}^{\circ }$	Gender	Chest-Wall Width [cm]	Height [m]	Distance D [cm]
1	Female	70	1.50	7
2	Male	94.1	1.69	16
3	Male	84.5	1.73	16.5
4	Female	74.5	1.65	11.5
5	Female	75.5	1.56	12
6	Male	82	1.60	13

## Data Availability

The data presented in this study are available on request from the corresponding author. The data are not publicly available due to privacy restrictions.

## Abbreviations

The following abbreviations are used in this manuscript:

CW	Continuous Wave
D	Distance
LHCP	Left Hand Circular Polarization
PCPTF	Pure Copper Polyester Taffeta Fabric
PET	Polyester Ethylene Terephthalate
RH	Relative Humidity
RX	Reception
SDR	Software Defined Radio
SEM	Scanning Electronic Microscope
SIGP	Substrate Integrating the Ground Plane
SIW	Substrate Integrated Waveguide
SNR	Signal-to-Noise Ratio
SMA	SubMiniature version A
TX	Transmission
UWB	Ultra-Wide Band
VNA	Vector Network Analyser
